# Resmetirom: A Systematic Review of the Revolutionizing Approach to Non-alcoholic Steatohepatitis Treatment Focusing on Efficacy, Safety, Cost-Effectiveness, and Impact on Quality of Life

**DOI:** 10.7759/cureus.69919

**Published:** 2024-09-22

**Authors:** Parikshit Bittla, Sai Pavitra Paidimarri, Shriya Ayuthu, Yashkumar D Chauhan, Moyal Z Saad, Amna A Mirza, Safeera Khan

**Affiliations:** 1 Internal Medicine, California Institute of Behavioral Neurosciences & Psychology, Fairfield, USA; 2 Medicine, California Institute of Behavioral Neurosciences & Psychology, Fairfield, USA

**Keywords:** fatty liver treatment, non-alcoholic fatty liver diseases (nafld), non-alcoholic steatohepatitis (nash), resmetirom, thyroid hormone receptor beta

## Abstract

There has been a rise in the prevalence of non-alcoholic steatohepatitis (NASH), a subset of non-alcoholic fatty liver disease (NAFLD) with an ongoing increase in the prevalence of linked conditions such as obesity, type II diabetes mellitus, and metabolic syndrome. To date, there are no specific drugs that are approved for the treatment of NAFLD/NASH. With the recent discovery of association between subclinical hypothyroidism and NASH, various trials exploring treatment options for NASH using thyroid hormone derivatives led to the discovery of resmetirom (MGL-316) with high affinity to thyroid hormone receptors (THRs) targeting the liver. Following standardized guidelines, a systematic review was performed on the safety, efficacy, and other practical aspects of resmetirom in the treatment of NASH. Advanced search was carried out using the MeSH search strategy and appropriate keywords in major databases using various inclusion and exclusion criteria. The search was narrowed down to seven high-quality articles: four randomized control trials (RCTs), and three reviews to be included in the current study. The online database search yielded 62 articles, out of which six high-quality articles were selected to be included in the current systematic review after deleting duplicates and screening for irrelevant titles, and articles. Out of the three RCTs, two of them assessed the safety and efficacy of resmetirom, while the remaining RCT assessed the impact on health-related quality of life with resmetirom on patients with NASH. resmetirom (MGL-316) is a thyroid hormone derivative with high affinity to THRs targeting the liver and acts by improving mitochondrial oxidation, and lipophagy in the hepatic cell line. All the trials suggested in favor of resmetirom with a decrease in NASH fibrosis score by at least two points, along with reduction in hepatic fat content (minimum relative reduction of 20%), liver volume by 61%, improving secondary outcomes such as low-density lipoprotein-C, apolipoprotein-B, triglycerides, and hepatic enzymes with greater reduction in the study groups treated with higher doses of resmetirom with no significant increase in adverse events. Resmetirom was found to improve patient-reported outcomes, and thereby quality-adjusted life years (QALYs) in 12 weeks while being cost-effective compared to placebo at a willingness-to-pay threshold of US$100,000 up to a daily threshold of US$72.00, and an effective incremental cost-effectiveness ratio of US$53,925 per QALY gained. After carefully analyzing the available data by our team members, it could be concluded that resmetirom holds a strong potential to be implemented as a drug of choice in treating NAFLD/NASH in the coming years with proven efficacy, safety while being cost-effective, and also reducing secondary co-morbidities by improving cardiovascular risk factors. The results can be best achieved when combined with conventional approaches such as weight loss and dietary modifications. Long-term safety and sustainability of the achieved results are yet to be confirmed with large-scale clinical trials. However, resmetirom is still an investigational drug and could be expected to be available for clinical practice in the near future.

## Introduction and background

Non-alcoholic fatty liver disease (NAFLD) management’s US guidelines defined NAFLD as steatosis with >5% fat infiltration in imaging or histology that is not alcohol/drug / viral-induced steatosis ranging from benign non-alcoholic fatty liver (NAFL) to non-alcoholic steatohepatitis (NASH) [1]. As said by Dr. Rohit Loomba, “NASH is like a ticking time bomb in the liver” and "it is the most common cause of chronic liver disease in the western world" as quoted by Dr. Quentin Anstee. It has always been linked to obesity, type II diabetes mellitus (DM), and metabolic syndrome in adults. With the ongoing increase in the prevalence of the conditions mentioned above, the incidence of NAFLD has been rising tremendously, with a worldwide prevalence of 25% [2]. NASH, a subset of NAFLD, is characterized by steatosis-associated inflammation and liver cell injury without any secondary cause, possibly leading to fibrosis, cirrhosis, and potential hepatic carcinoma [3]. It has been identified that the development of NASH is a two-step process. In the first stage, fat deposited in the liver will increase insulin resistance, followed by cellular and molecular changes leading to fatty acid oxidation and inflammation [4].

The symptoms of NASH can include right upper quadrant discomfort, fatigue, hepatomegaly, and acanthosis nigricans. However, more than half of the patients remain asymptomatic and are often discovered while undergoing evaluations for other medical conditions [5]. The laboratory studies in individuals with NASH may reveal mild to moderate elevation in hepatic transaminases, with alanine transferase (ALT) levels being higher than aspartate transferase (AST) levels in the majority of the cases [6]. Ultrasonography is commonly used to aid in diagnosis, and it often reveals a hyperechoic texture or diffuse fatty infiltration of the liver. However, biopsy remains the gold standard for diagnosing NASH demonstrating inflammation and balloon degeneration in addition to steatosis [7].

The conservative management of NASH primarily focuses on lifestyle modifications and weight loss. Studies suggest that the Mediterranean diet, with omega-3 fatty acid intake, has significantly reduced liver fat. Adding vitamin E, caffeine, and other antioxidants can be beneficial in the management of NASH [8-10]. Due to serum insulin levels and biochemical alterations of liver enzymes in NASH, weight loss is considered the primary therapy for most patients [11]. To date, there are no specific drugs that are approved for the treatment of NAFLD/NASH, and a lot of trials have been carried out to evaluate the efficacy and safety of investigational drugs. Several studies have shown that the use of pioglitazone and glucagon-like peptide-1 (GLP-1) for weight loss (by increasing insulin sensitivity) can improve the biochemistry of liver enzymes significantly [12,13]. In recent years, bariatric and metabolic surgery has gained popularity in treating obesity, which could in turn aid in reducing hepatic fat content [14].

However, the recent discovery of an association between subclinical hypothyroidism and NASH has led to various trials exploring the role of thyroid hormones in NASH pathophysiology and treatment. Resmetirom (MGL-316) is one such thyroid hormone derivative with high affinity to thyroid hormone receptors (THRs) targeting the liver and was shown to be the most effective among other derivatives in the treatment of NASH in recent times. The majority of the systematic reviews described the mechanism of action of resmetirom along with other pharmacological properties. However, this review explores the role of thyroid hormone derivates in NASH along with a focus on the effect of resmetirom in patients with NASH while considering the aspects of safety, affordability, and quality of life. 

## Review

Methods and search strategy

Six articles were included in the study after performing a comprehensive search of various electronic databases for relevant data on the use of thyroid receptor agonists in the treatment of NASH using appropriate keywords and Medical Subject Headings MeSH - ‘Resmetirom’, ‘Thyroid Hormone Receptor Beta Agonists’, ‘Non-Alcoholic Steato-Hepatitis’, ‘Non-Alcoholic Fatty Liver Disease’ [15-20]. We conducted our search in the major databases PubMed, PubMed Central, and MEDLINE. The above search was carried out following strict guidelines of Preferred Reporting Systems for Systematic Reviews and Meta-Analysis [21]. Our final search strategy includes

1. ( "Thyroid Hormone Receptors beta/administration and dosage"[Mesh] OR "Thyroid Hormone Receptors beta/agonists"[Mesh] OR "Thyroid Hormone Receptors beta/antagonists and inhibitors"[Mesh] OR "Thyroid Hormone Receptors beta/classification"[Mesh] OR "Thyroid Hormone Receptors beta/deficiency"[Mesh] OR "Thyroid Hormone Receptors beta/drug effects"[Mesh] OR "Thyroid Hormone Receptors beta/therapeutic use"[Mesh] )

2. ( "Non-alcoholic Fatty Liver Disease/classification"[Mesh] OR "Non-alcoholic Fatty Liver Disease/complications"[Mesh] OR "Non-alcoholic Fatty Liver Disease/drug therapy"[Mesh] OR "Non-alcoholic Fatty Liver Disease/prevention and control"[Mesh] OR "Non-alcoholic Fatty Liver Disease/therapy"[Mesh] ).

The above MeSH strategy was combined with an ‘AND’ in advanced search to obtain relevant articles using the following filters: articles published from 2019 to 2024, articles written in English language, and full-text free articles and the full search strategy is presented in Table 1. The search result was carried out by two personnel. Articles obtained from PubMed Advanced using the MeSH strategy were exported to Excel Sheet after transferring to EndNote Basic (Clarivate, Philadelphia, US). Additional papers were recruited from further databases such as MEDLINE, and PubMed Central using the search words ‘Resmetirom’, ‘Thyroid Hormone Receptor Agonists’, ‘Thyroid Hormone Receptor Beta Agonists’, ‘Non-Alcoholic Fatty Liver Disease’, Non-Alcoholic Steato-Hepatitis’. The obtained articles were screened for irrelevant titles not related to the topic of interest. We went thoroughly through the remaining articles for abstract and full-text screening for inclusion of the content only relevant to the research question. Papers not focusing on the topic of interest were discarded. The final articles were evaluated for inclusion into study using standard quality assessment tools.

**Table 1:** Search Strategy RCT: Randomized Control Trial

Database	Search Strategy	Characteristics
PubMed PubMed Central	(( "Thyroid Hormone Receptors beta/administration and dosage"[Mesh] OR "Thyroid Hormone Receptors beta/agonists"[Mesh] OR "Thyroid Hormone Receptors beta/antagonists and inhibitors"[Mesh] OR "Thyroid Hormone Receptors beta/classification"[Mesh] OR "Thyroid Hormone Receptors beta/deficiency"[Mesh] OR "Thyroid Hormone Receptors beta/drug effects"[Mesh] OR "Thyroid Hormone Receptors beta/therapeutic use"[Mesh] )) AND (( "Non-alcoholic Fatty Liver Disease/classification"[Mesh] OR "Non-alcoholic Fatty Liver Disease/complications"[Mesh] OR "Non-alcoholic Fatty Liver Disease/drug therapy"[Mesh] OR "Non-alcoholic Fatty Liver Disease/prevention and control"[Mesh] OR "Non-alcoholic Fatty Liver Disease/therapy"[Mesh] ))	Timeline : 2019-2024, Articles written in English, Observational Studies, RCTs, Systematic Reviews, Meta-Analysis
MEDLINE	Keywords: ‘Resmetirom’, ‘Thyroid Hormone Receptor Agonists’, ‘Thyroid Hormone Receptor Beta Agonists’, ‘Non-Alcoholic Fatty Liver Disease’, Non-Alcoholic Steato-Hepatitis’	Timeline: 2019-2024, Articles written in English Observational Studies, RCTs, Systematic Reviews, Meta-Analysis

Inclusion and Exclusion Criteria 

The literature must be written in English, later than 2019 with content relevant to the topic of interest in order to be included. Only original articles, clinical trials, observation studies, systematic reviews, and traditional reviews are considered. Any editorials, comments, perspectives, peer reviews, unpublished studies, and animal studies were excluded from the review. The criteria for inclusion and exclusion are presented in Table 2.

**Table 2:** Inclusion and Exclusion Criteria RCT: Randomized Control Trial; NASH: Non-alcoholic Steatohepatitis

Characteristics	Inclusion Criteria	Exclusion Criteria
Language	Articles published in English language	Articles published in languages other than English
Type of Study	Observational studies, RCTs, Systematic Reviews, Traditional Reviews, Meta-Analysis.	Editorials, Perspectives, Case Studies, Peer Reviews, Commentaries, Unpublished Studies, and Animal Studies
Year of Publishing	Articles published after 2019	Articles published before 2019
Content of Study	Articles with content focusing on the topic of interest	Articles with content irrelevant to the research question of interest
Disease Status of Study Participants	Individuals must be diagnosed with NASH prior to study	Individuals without NASH were excluded from the study

Analysis of Quality of Study/Bias

Twelve articles were finalized for quality assessment after deleting duplicates, excluding titles irrelevant to the topic of interest, and abstract, full-text screening of the articles obtained via above mentioned electronic databases. Among the 12 articles, six were categorized as high or medium-quality papers and were included in the study [15-20]. The quality assessment tools utilized in analysis of articles are: a) the Jadad scale for Randomized Control Trials (RCTs) and b) The Scale for the Assessment of Narrative Review Articles (SANRA) criteria for Traditional Reviews which are represented in Tables 3, 4.

**Table 3:** Jadad Scale for Randomized Control Trials

Jadad Scale	Harrison et al. 2021 [15]	Harrison et al. 2023 [16]	Younosi et al. 2021 [17]
1. Was the method of randomization adequate?	yes	yes	yes
2. Was the treatment allocation concealed?	yes	yes	yes
3. Were study participants and providers blinded to treatment group assignment?	yes	yes	yes
4. Were the people assessing the outcomes blinded to the participants' group assignments?	no	yes	no
5. Were the groups similar at baseline on important characteristics that could affect outcomes?	yes	yes	yes
6. Was the overall drop-out rate from the study at endpoint 20% or lower of the number allocated to treatment?	yes	no	yes
7. Was the differential drop-out rate at endpoint 15 percentage points or lower?	yes	yes	yes
8. Was there high adherence to the intervention protocols for each treatment group?	yes	no	yes
9. Were other interventions avoided or similar in the groups?	yes	yes	yes
10. Were outcomes assessed using valid and reliable measures, implemented consistently across all study participants?	yes	yes	yes
11. Did the authors report that the sample size was sufficiently large to be able to detect a difference in the main outcome between groups with at least 80% power?	no	yes	no
12. Were all randomized participants analyzed in the group to which they were originally assigned, i.e., did they use an intention-to-treat analysis?	yes	yes	yes
Assigned Score	10/12	10/12	10/12

**Table 4:** The Scale for Assessment of Narrative Review Articles (SANRA) Checklist for Traditional Reviews

SANRA Checklist	Javanbakth et al. 2023 [18]	Karim et al. 2023 [19]	Zhao et al. 2022 [20]
Justification of article's importance	2	2	2
Concrete aims / formulation of questions	2	2	2
Description of literature search	0	0	0
Referencing of key statements	2	2	2
Scientific reasoning / appropriate evidence	2	2	2
Appropriate representation of data	2	2	2
Assigned Score	10/12	10/12	10/12

Results 

**Figure 1:**
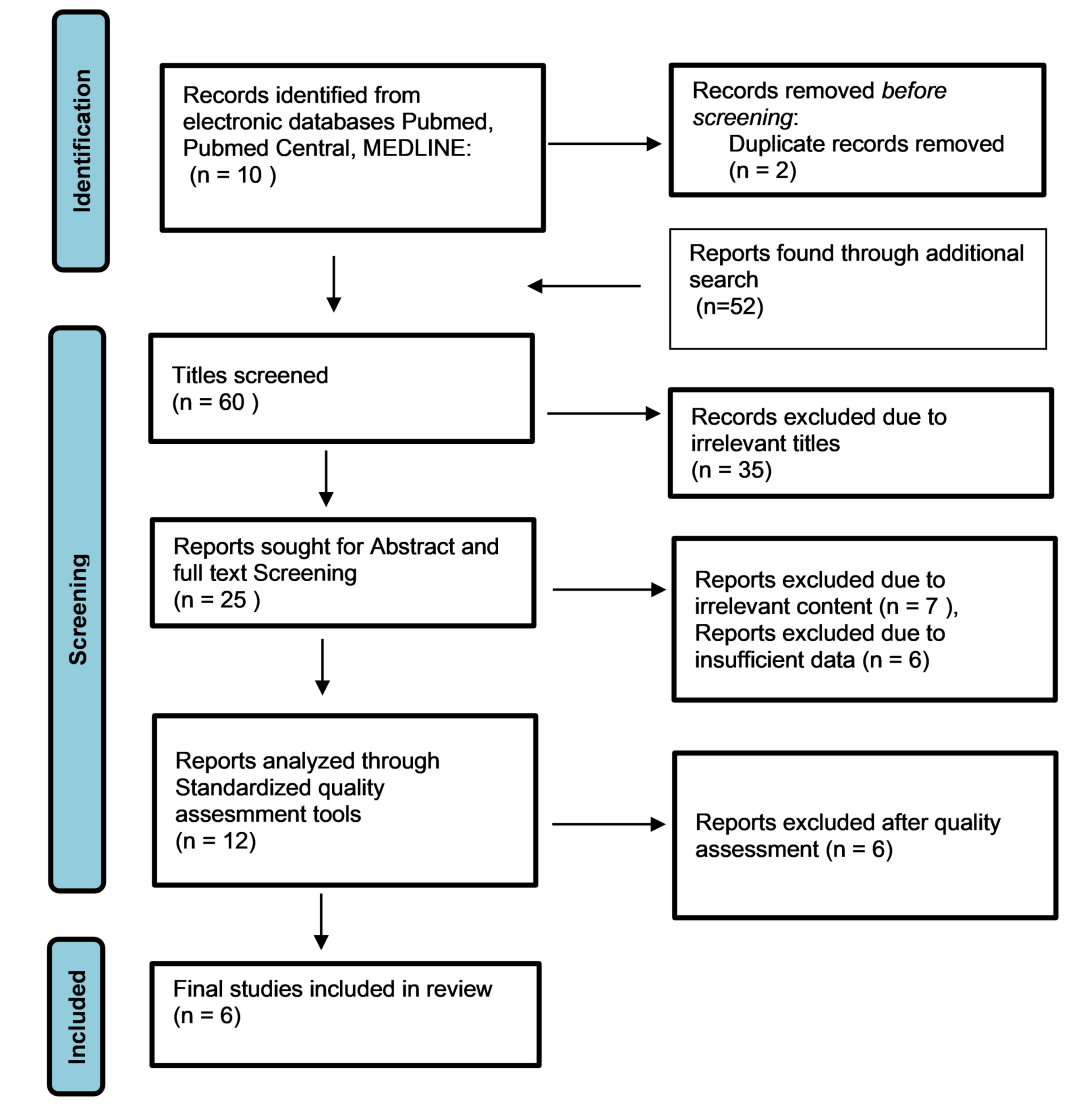
Preferred Reporting Systems for Systematic Reviews and Meta-Analysis Flowchart Source: [21].

Sixty-two papers were obtained from the electronic database search as a part of the search strategy. After papers were subjected to title screening, 25 articles underwent full-text screening and 12 were qualified for quality assessment. In formulating this systematic review, six high-quality articles were finalized and included. The search results are presented in Figure 1. The finalized articles consist of three randomized control studies and three traditional reviews including 2272 patients with characteristics of included studies presented in Table 5. The results of RCTs revealed that resmetirom improved the NASH score by at least two points, and fibrosis of the liver by one point with no associated worsening of fibrosis. In addition, it also reduced the liver volume (average reduction of 61%), liver stiffness (32-55% in the resmetirom arms versus 25% in the placebo arm), and 100% of patients achieving at least 20% relative reduction in hepatic fat content from baseline. Low-density cholesterol (LDL-C), apoprotein B, apolipoprotein C, triglycerides (TGs), hepatic enzymes, and other secondary outcomes were also found to be lowered with resmetirom treatment. The above improvements were found to be higher with increased doses of resmetirom (80mg and 100mg) and there was no increase in adverse events or worsening of fibrosis as compared to placebo with higher doses. Patients who were treated with resmetirom have reported significant improvement in comparison to placebo in various patient-reported outcomes such as physical component summary (+2.99 ± 0.76; P = 0.00062), mental component summary (+10.3 + 3.9; P = 0.018), and bodily pain (+9.39 ± 3.18; P = 0.010), thereby improving quality adjusted life years (QALYs) by the end of 12 weeks which continued until week 36 of trial. The average cost of resmetirom per person was estimated to be around US$66,764. The drug was proven to be cost-effective compared to placebo at a willingness to pay threshold of US$100,000 up-to a daily threshold of US$72.00 with an effective incremental cost-effectiveness ratio of US$53,925 per QALY gained.

**Table 5:** Characteristics of Included Studies NAFLD: Non-alcoholic Fatty Liver Disease; NASH: Non-alcoholic Steatohepatitis; TAE: Treatment-Related Adverse Events; HRQL: Health-Related Quality of Life; QALY: Quality Adjusted Life Years; THR: Thyroid Hormone Receptor; MRI-PDFF: Magnetic Resonance Imaging-Proton Density Fat Fraction; CV: Cardiovascular; TR: Thyroid Receptors: LDL-C: Low-Density Lipoprotein C

Study	Year of Study Published	Type of Study	Population of Study	Aim of Study	Results/Outcomes
Harrison et al. [15]	2021	Randomized Control Trial - Phase II	38	Impact of 80 and 100mg daily doses of resmetirom on safety and noninvasive assessments of NASH	Resmetirom has reduced the MRI-PDFF significantly in patients with biopsy-confirmed NASH along with other related parameters (LDL, Apo-B, TGs) supporting efficacy and safety of resmetirom
Harrison et al. [16]	2023	Randomized Control Trial – Phase III	1143	Assessing safety and tolerability of 80mg and 100mg resmetirom vs placebo in patients with NAFLD/NASH as measured by incidence of TAEs over 52 weeks of study	Resmetirom was well tolerated at both 80mg and 100mg doses over 52 weeks with no increase in serious TAEs
Younossi et al. [17]	2021	Randomized Control Trial	125	Impact on HRQL with use of resmetirom using self-administered Short Form-36 in non-cirrhotic patients with NASH	Resmetirom leads to improvement in HRQL especially in Physical Functioning, Vitality, and Mental Health Domains in patients with NASH
Javanbhakt et al. [18]	2022	Systematic Review		Assessment of relative cost-effectiveness of resmetirom as compared to placebo alongside a pricing analysis	Resmetirom is a potentially cost-effective option for patients with NASH and liver fibrosis at a willingness-to-pay threshold of US $100,00 up to a daily price point of US$72.00 with an incremental cost-effectiveness ratio of US$53,929 per QALY gained.
Karim et al. [19]	2023	Systematic Review		Exploring the role of THR-β agonists in etiology and treatment of NASH with focus on Resmetirom	Resmetirom is effective in reducing hepatic fat content as measured by MRI-PDFF, liver enzymes with improvement in non- invasive markers of liver fibrogenesis, decreased liver stiffness while contributing to favorable CV profile by reducing serum lipids
Zhao et al. [20]	2022	Systematic Review		Development of thyroid hormones and thyromimetics based on TR selectivity for NAFLD, analyzing the role of TR-targeted drug in the treatment of NAFLD	Resmetirom (MGL-3196) is found to be the most promising TRβ-selective agonist for NASH treatment with potential to reduce plasma LDL-C and triglyceride concentrations accompanied by cardio-protective potential

Discussion

Role of Thyroid Hormones in NASH

The pituitary gland releases thyroid-stimulating hormone (TSH) that promotes the release of hormones T3 and T4 from the thyroid gland. The released T4 is converted into the active metabolite T3 peripherally by the enzyme deiodinase-1. It is hypothesized that this conversion of proactive T4 into active T3 is decreased in the liver of patients with NASH, suggesting the critical role of T3 in NAFLD/NASH [22]. The effects of T3 peripherally are mediated through two subtype receptors, thyroid hormone receptor-α (THR-α) expressed in the heart, brain, and skeletal muscle and Thyroid Hormone Receptor-β (THR-β) expressed in the liver. The role of thyroid hormones was regulated by the receptor THR-β in the liver [23-25]. By inducing dose-dependent autophagy in hepatoma cell line Hep G2 and promoting lipophagy, T3 delivers the free fatty acids for mitochondrial beta-oxidation, explaining the mechanism through which thyroid hormones promote lipid homeostasis through autophagy and mitochondrial oxidation due to mitophagy and mitochondrial biogenesis [26]. With the discovery that thyroid hormone (TH) signal change in cells can be a possible factor in developing liver-related diseases such as NAFLD, the role of thyroid hormones has been constantly studied for the treatment of NASH, which is a subset of NAFLD. The review conducted by Karim et al. stated that as the levels of serum TSH increase, the prevalence of NASH increases significantly after adjusting for age, gender, and smoking status [19]. Though T3 can target the THR-β in the liver, its high affinity for THR-α in the cardiac cells can lead to severe adverse reactions. Hence, there was an unmet need to find a drug that selectively targets THR-β, and many thyromimetics have been studied that have the potential to treat NASH. 

The role of thyroid hormones and their derivatives were explored by Karim et al. and Zhao et al. in a traditional review discussing the following molecules and their role in NASH [19,20]. The study done by Mollica et al. found that T2 (a metabolite derived from T3 in the presence of deiodinase-2 with 50-1000 times lower affinity to thyroid receptor (TR) than T3) reduced fat accumulation and increased the oxidation of fatty acids at the mitochondrial level, thereby significantly reducing the steatosis of the liver in the lab rats [27]. However, T2 was not extensively studied due to its lower affinity to THRs. Another biogenic amine, 3-iodothyronine (T1AM), is an endogenous metabolite acting on trace amine-associated receptor-1 (TAAR1) rather than TR receptors, with T3 antagonism distributed in the liver, brain, and muscle tissues with a high concentration in the liver [28]. T1AM administration in rats altered liver-related gene expression profile with increased lipid decomposition and oxidation [29]. The use of T1AM is yet to be confirmed for the treatment of NAFLD/NASH. In subsequent clinical trials, thyroid hormone drugs called synthetic thyromimetics have been developed over decades, from GC-1, the initial thyroid hormone drug, to the latest and most successful MGL-3196 [30,31]. GC-1 (3,5-Dimethyl-4 (4′-hydroxy-3′-isopropylbenzyl)-phenoxy) acetic acid), also known as Sobetiromeis the first synthetic thyroid hormone drug with similar selectivity to receptor TRβ when compared to T3 with a 10-fold lower affinity to TRα1 and thereby enhanced therapeutic effect with lower rates of adverse events. In addition to reducing cholesterol and triglyceride levels, it also exhibited elimination of hepatic pre-neoplastic lesions. However, the administration of GC-1 has led to increased fasting blood sugar levels and insulin resistance in the model rats, and hence, it was discontinued [32].

Later, a second-generation TR-selective agonist, GC-24, a derivative of GC-1, was developed with 40-fold higher affinity to TRβ compared to that of TRα, which significantly improved plasma triglyceride level, glucose tolerance, and insulin sensitivity but was unable to restore the increased hepatic cholesterol content, hypercholesteremia and had a poor liver target than GC-1 or T3 [33,34]. Subsequently, KB141 and KB2115 TR selective molecules with a 10-fold higher affinity to thyroid receptor β than thyroid receptor α reducing cholesterol, lipoprotein, triglycerides, and fatty acids have been reported. Though there is no application of KB141 in NAFLD/NASH, KB2115 induced net cholesterol excretion with a 40% reduction in total and low-density lipoprotein-C (LDL-C) in two weeks. A 12-week phase II trial was conducted where it reduced LDL-C, triglyceride, and atherosclerotic lipoprotein in patients who are on statin in human trials and prevented liver steatosis in animal models [35,36]. However, due to adverse effects of KB2115 noted on dogs' cartilage after long-term administration, the phase III clinical trial was terminated. Other TR-selective agonists MB07344 and MB07811 significantly reduced cholesterol and triglycerides in the liver of the diet-induced obese mice model. MB07811 has exhibited a dramatic effect of ameliorating hepatic steatosis and also cleared the lipid levels by increasing differential expression of hepatic genes, increased mitochondrial respiratory rate, and plasma acyl-carnitine levels. MB07811 is currently renamed VK2809 and is undergoing testing in phase-II trial.

Recently, MGL-3196 Resmetirom has gained popularity due to its unique ability to treat NASH. It is a TR selective agonist 2-(3,5-dichloro-4-(5-isopropyl-6-oxo-1,6-dihydropyridazin-3-yloxy) phenyl)-3,5-dioxo-2,3,4,5-tetrahydro(1,2,4) triazine-6-carbonitrile, with cyanoazauracil substituent introduction improving the efficacy, and selectivity of THR with a 28 fold higher selectivity for thyroid receptor β than α significantly and no cardiac adverse events, does not alter the central thyroid axis [19,20]. Most of the drug (99%) of the drug is bound to protein. Hence, penetration outside the liver is poor, making the liver the specific target of the drug [15]. MGL-3196 showed strong potential in treating NASH in various clinical trials by lowering LDL-C, triglycerides, and lipoprotein A levels with a favorable side effect profile. The first clinical data on resmetirom in 72 healthy human populations was a single-center, randomized, double-blinded, placebo-controlled trial assessing the pharmacokinetics and safety of the drug, along with a two-week multiple-dose study with doses ranging from 5mg to 200mg in 48 subjects with mildly elevated LDL of >110mg/dl assessing its impact on thyroid axis, and LDL-lowering effects. The first seminal clinical trial was a 36-week, phase II study in biopsy-confirmed NASH individuals of fibrosis stages 1-3 with serial liver biopsies determining the ability of resmetirom in reducing the lipotoxic lipids that are causative agents of hepatotoxicity using Magnetic Resonance Imaging proton density fat fraction (MRI-PDFF) fat quantification. Subsequently, the open-label extension (OLE) study as an open-label extension of the phase III trial and MAESTRO-NASH trial was designed to assess the effects of resmetirom in a real-world setting.

Resmetirom vs NASH

In 2021, a 36-week multi-centered randomized double-blinded, placebo-controlled phase II 2:1 clinical trial was conducted by Harrison et al. on 38 subjects with biopsy-confirmed NASH (fibrosis stages 1-3 with a minimum hepatic fat fraction of 10%) and followed up with serial hepatic fat measurements assessed by MRI-PDFF, where patients were randomized to a placebo group or resmetirom (60 mg) group [15]. In the subset of patients with incomplete responses (liver enzymes not fully normalized at the end of the study-worsening values with > 30% from baseline and greater than the upper limit of normal or improved but elevated levels > 1.5-2 fold of the upper limit or improved or worsened with > two times the upper limit), an active treatment open-label extension (OLE) analysis was conducted in 31 out of 38 eligible patients where the dosage of resmetirom was increased (Res/Res) or switched to resmetirom from the placebo (Pbo/Res) and assessed the outcome of 80mg or 100mg daily doses of resmetirom. The NASH activity score (NAS) at week 36 showed a two-point reduction of NAS in 53% of patients whose resmetirom dose was increased in the OLE study, and a reduction in fibrosis was seen in around 17.6% of biopsies as compared to 14.3% reduction in two-point NAS and 0% fibrosis reduction in the patients who were switched from placebo to resmetirom.

The results of this trial demonstrated that higher doses of resmetirom resulted in a more significant reduction of hepatic fat content on week 12 MRI-PDFF compared to baseline, with a higher incidence of NASH resolution and fibrosis reduction. However, the small sample size precluded a comparison between the 80 mg and 100 mg doses. The average reduction in PDFF during the main study in the subjects whose resmetirom dose was increased in the OLE study at week 36 was 27.9%, and at week 12 of the OLE study was 31.6%, which did not change significantly from the main study. However, after week 12, when dose increments were made, both the arms Pbo/Res and Res/Res (80 mg) experienced a mean relative reduction of 52.3% + 4.4% (P<0.0001), with an absolute reduction of 11.1% + 1.5%. Patients who received a 100 mg dose of resmetirom have experienced a higher mean relative reduction of 58.8% + 6.8%, with an absolute reduction of 14.3% + 1.9%. Around 85% of the patients receiving 100 mg experienced a minimum of 30% relative reduction in PDFF, with 100% of patients achieving at least 20% relative reduction with a minimum 5% absolute reduction. However, the controlled attenuation parameter (CAP) score, a component of the FibroScan measurement (a marker of hepatic steatosis), showed no significant correlation with PDFF at week 36 and was not found to be reduced with the use of resmetirom.

Following the promising results of the Phase II trial, Harrison et al. conducted the Phase III Randomized Control Trial MAESTRO-NAFLD-1 at 80 sites in the United States from 2019 to 2021. The trial encompassed 1,143 patients, with 972 randomly assigned to one of three double-blinded arms: 100 mg resmetirom (n = 325), 80 mg resmetirom (n = 327), or placebo (n = 320). Additionally, we randomized 171 patients to the open-label 100 mg resmetirom, with the primary objective being to evaluate the safety and tolerability of both 80 mg and 100 mg resmetirom in patients with NAFLD/NASH. The study evaluated the impact on LDL-C levels along with other parameters such as hepatic fat content, apolipoprotein-B, and triglycerides as secondary outcomes. The study found that the amount of hepatic fat dropped significantly. It dropped from -47.8% (-53.8% to -41.8%) in the open-label 100 mg resmetirom group to -45.1% (-50.3% to -39.9%) in the double-blinded 100 mg resmetirom group to -41.4% (-46.6% to -36.2%) with 80 mg resmetirom compared to just 6.5% with a placebo at week 16, and the drop lasted for 52 weeks with continued treatment (P<0.0001 versus placebo for all three comparisons). At week 52, the least squares mean relative reduction from baseline was -51.8% (-58.6% to -45.0%) with 100 mg open-label resmetirom [16]. At week 52, the MRI-PDFF showed that patients who had been treated with resmetiorm had less fat in their livers in all patient groups, but especially in those whose body weight had dropped by at least 5% from the start. Additionally, patients on resmetirom showed a more significant reduction in the liver stiffness measurement of >2kPa from baseline, as assessed by vibration-controlled transient elastography (VCTE), with 32-55% in the resmetirom arms and 25% in the placebo arm. Also, more people in the resmetirom group (22-25%) than in the placebo group (11%) were able to reduce their liver stiffness by more than 19%, as measured by magnetic resonance elastography (MRE). Resmetirom treatment reduced the liver volume by 21% and 23% from baseline at weeks 16 and 52 weeks, respectively. Correcting for the reduced liver volume led to an average reduction of around 61% in the open-label resmetirom arm (P<0.001).

Harrison et al. conducted another ongoing phase III trial in adults with biopsy-confirmed NASH in stages F1B, F2, and F3 of fibrosis, who received a daily dose of 80 mg resmetirom, 100 mg resmetirom, or placebo in a 1:1:1 ratio [37]. The primary endpoints were a reduction in NAFLD activity score by >two points and an improvement in fibrosis by at least one stage. In the 80 mg resmetirom group, 25.9% of the subjects achieved NASH resolution with no associated worsening of fibrosis. In the 100 mg resmetirom group, 29.9% of individuals achieved the results, compared to 9.7% in the placebo group (P<0.001 for both comparisons with placebo). Patients with > 30% hepatic fat reduction showed an increased rate of NASH resolution (37%) and improved patient-related outcomes (PROs). Fibrosis was improved by at least one stage in 24.2% of the patients in the 80 mg resmetirom group and 25.9% in the 100 mg resmetirom group compared to 14.2% in the placebo (P<0.001 for both comparisons with the placebo). The 2021 trial by Younossi et al. also revealed similar results, with a 30% reduction in proton density fat fraction by the end of week 12. It was associated with improved physical functioning and physical component summary (PCS) scores [17]. Patients treated with a higher dose of resmetirom experienced a more significant reduction in the hepatic fat at week 12. At week 36, they had a higher incidence of NASH resolution and liver fibrosis. Resmetirom proved to be highly effective in all the trials mentioned above, with the resolution of NASH directly proportional to the drug's dosage.

Secondary Outcomes

The phase II trial by Harrison et al. found that resmetiorm treatment lowered LDL-C by up to 26.1% + 4.5% (P<0.0001), apolipoprotein B (-23.8% + 3.0%, P<0.0001), apolipoprotein C-III (_21.6% + 3.7%, P<0.0001), and triglycerides (-46.1% + 14.5%, P = 0.0036) compared to the start of the study. The study also evaluated the levels of liver enzymes, revealing a decline over time in alanine aminotransferase (ALT) by 23.3% + 6.7% (P = 0.00016) and glutamyl transferase (GGT) by 24.4% (P = 0.0006), accompanied by an increase in sex hormone-binding globulin (SHBG), believed to be the biomarker of resmetiorm activity. Both the phase II main study and the OLE study assessed the parameter N-terminal pro-peptide of type III collagen (PRO-C3), which is considered the liver regression biomarker. The primary survey showed an increase in C3M levels, indicating a regression of fibrosis, while the placebo group showed no significant change. The OLE study with resmetirom treatment significantly reduced the levels of PRO-C3/C3M, a marker of fibrosis formation (P = 0.0044). The OLE study with resmetirom significantly improved Adiponectin, another potential marker of liver fibrosis, and also reduced liver stiffness on FibroScan (2.1 [0.8] kPa, P = 0.015). The ongoing phase III trial of Harrison et al. showed comparable results, with a 13.6% decrease in LDL-C levels from baseline to week 24 in the 80 mg resmetirom group. The 100 mg group showed a 16.3% reduction when compared to the placebo, whereas the reduction was seen only in 0.1% of the subjects (P<0.001 for both comparisons with placebo) [15].

The MAESTRO-NAFLD-1 trial also looked at how much LDL-C, apolipoprotein B and triglycerides dropped over 24 weeks, as well as liver fat over 16 and 52 weeks and liver stiffness over 52 weeks [16]. The difference in reduction from placebo of given levels in individuals treated with 80mg or 100mg resmetirom is as follows: LDL-C (−11.1%, −12.6%), apolipoprotein B (−15.6%, −18.0%), triglycerides (−15.4%, −20.4%), 16-week hepatic fat (−34.9%, −38.6%), (P < 0.0001), liver stiffness (−1.02, −1.70) and 52-week hepatic fat (−28.8, −33.9) where increased reduction was noted with the higher dose of resmetirom. The 100 mg resmetirom significantly reduced LDL-C from baseline compared to the placebo (-13.9%), while also reducing apolipoprotein B by 16.5% and triglycerides by 23.4% from the baseline. Lowering LDL-C, apolipoprotein B, and triglyceride levels from the start was better with 80 mg resmetirom than with placebo, but not as good as with 100 mg dose resmetirom. The drops were 12.4%, 14.3%, and 18.4%, respectively. In the meantime, the open-label 100 mg resmetirom arm demonstrated reductions in LDL-C, apolipoprotein B, and triglyceride levels of 19.4%, 21.3%, and 27.5%, respectively. These reductions were more significant than the levels attained in the 100 mg resmetirom arm at week 24, potentially due to missed doses in the 100 mg double-blinded resmetirom arm due to the COVID-19-related delay in drug kit delivery. Continued treatment maintained all the above-achieved results for 48 weeks, demonstrating resmetirom's potential to successfully improve secondary outcomes in NASH patients, aiding in NASH resolution and reducing cardiovascular outcomes. However, long-term follow-up is required to confirm the results' sustainability, and, thus, the mortality benefit from decreasing complications.

ALT, AST, and gamma-glutamyl transferase (GGT) levels, which are signs of liver damage, were found to be significantly lower from the start in the open-label 100 mg resmetirom, double-blinded 100 mg resmetirom, and 80 mg resmetirom groups compared to the placebo groups at week 52 of the MAESTRO-NAFLD-1 trial (P<0.05 versus placebo for all) [16]. Owing to the increased activation of THR β receptors in the liver, Sex Hormone Binding Globulin (SHBG) gradually increased until week 12. At week 24, it reached a plateau that corresponded to the reduction in ALT/AST.

Researchers found that type III collagen is a critical component of liver fibrosis in NASH patients, necessitating the identification of non-invasive, reliable measures to estimate the progression of fibrosis. We can assess the levels of PRO-C3 and C3M serum markers to determine the progression of fibrosis, as they reflect the formation and degradation of type II collagen. When treated with resmetirom, PRO-C3, and C3M levels decreased by 0.68% (P<0.0001). Additionally, the administration of resmetirom decreased liver stiffness, a marker of fibrosis, by 0.76% (P=0.0044) [15].

Effect of Resmetirom on the Thyroid Axis

In the phase II trial by Harrison et al. in 2021, there were no significant differences in baseline levels of free T4 (FT4) or thyroid stimulating hormone (TSH) between people with NASH and age-matched people who did not have NASH. In the OLE study [15], there was a mild decrease of 10.9% (P<0.05) in FT4. The MAESTRO-NAFLD-1 trial's results, which showed minimal reductions in FT4 levels from baseline with resmetirom but no effects on FT3 or TSH, confirmed the findings [16]. But reverse T3 (rT3), which is a sign of inflammation in the liver, was higher in people with NASH, and the ratio of FT3/rT3 was lower (P<0.0001). As fibrosis increased, the ratio declined. When resemetiorm was used, rT3 levels dropped a lot, and the ratio of FT3/rT3 went up in both primary and OLE studies. This shows that resemetirom has greatly reduced liver inflammation while improving fibrosis [15]. Other studies included in this review did not evaluate the drug's impact on the thyroid axis, although they reported any adverse events of disturbed thyroid hormonal parameters during the trials.

Adverse Events and Safety of Resmetirom

The phase II Harrison et al. trial of 2021 and the OLE study both found that the resmetirom group did not have any serious side effects. In fact, only 18 of the 31 people in the treatment arm (58 percent in the resmetirom vs. 68 percent in the placebo arm) reported mild (26 percent) to moderate (32 percent) events. And 10% of individuals reported diarrhea as the most common complaint, followed by headache, urinary tract infection, and dizziness, suggesting fewer adverse events with resmetirom. Additionally, patients in the treatment arm showed no elevation in liver enzymes, while approximately 19% in the placebo group showed elevated enzymes (ALT and GGT were five times the upper normal limit in 7% and 12% of the individuals, respectively). The MAESTRO-NAFLD trial found that treatment-emergent adverse events (TAEs) happened in 86.5% of patients in the open-label 100 mg resmetirom group, 86.1% of patients in the 100 mg resmetirom group, 88.4% of patients in the 80 mg resmetiorm group, and 81.8% of patients in the placebo group over the course of 52 weeks. There was no significant association of TAEs with resmetirom compared to the placebo group. COVID-19 pneumonia accounted for around 20% of the reported incidences of adverse events. However, the resmetirom group experienced more frequent adverse events, diarrhea, and nausea than the placebo-treated groups (23.5-32.2% in resmetirom arms versus 13.8% in placebo, 11.9-18.2% in resmetirom arms versus 7.9% in placebo arms, respectively), particularly during the first 12 weeks of treatment. The incidence of diarrhea and nausea was similar to that of the placebo group after 12 weeks of trial, with the median duration of diarrhea being 15 to 20 days in resmetirom groups independent of the dosage. Approximately 50% of the diarrhea cases were either a single episode, intermittent diarrhea, or a worsening of the underlying diarrhea. The incidence of diarrhea was similar in both sexes, while the nausea was more frequent in females than males, including the placebo arms. Harrison et al. conducted an ongoing RCT and found that the incidence of adverse events was similar in the trial groups: 10.9% of subjects in the 80 mg resmetirom group experienced adverse reactions, and 12.7% in the 100 mg group, compared to 11.5% in the placebo group, concluding that the side effect profile of resmetirom was favorable and comparable to that of placebo [37].

Quality of Life With Resmetirom

NAFLD/NASH was known to decrease the health-related quality of life (HRQL), work productivity and impair patient-reported outcomes (PROs) in addition to the clinical burden contributing to increased cardiovascular risk and other health-related complications [38]. Hence in 2021, a phase II multicenter Randomized Control Trial was conducted by Younossi et al. in 125 biopsy-proven non-cirrhotic NASH patients with a minimum hepatic fraction of at least 10% in MRI-PDFF to assess the impact on HQRL in patients treated with 80mg resmetirom or placebo whose baseline HRQL scores were not different from the general population norms using self-administered Short Form-36 (SF-36) [17]. The patients filled out the SF-36 form before the start of the treatment process and then re-assessed every 12 weeks until the study week 36 using eight domains - Physical Functioning, Role Physical, Bodily Pain, General Health, Vitality, Social Functioning, Role Emotional, and Mental Health ranging from 0-100 along with two summary scores: physical component summary (PCS) and mental component summary (MCS). PROs are essential to the clinical trial process, providing critical perspectives on patients' experience with the disease or treatment. When the patients were treated with resmetirom, by the end of 12 weeks, they experienced improved PCS and continued to improve up to week 36 of treatment (+2.99 + 0.76; P = .00062). By the end of week 36, PCSS was improved in patients of the resmetirom group (+1.40 ± 0.40; P = .030), while no such improvement was seen in the placebo arm. Also, Patients who received resmetirom experienced an improved bodily pain domain score (+6.31 + 2.67; P=0.022) and Short Form-6D (SF-6D) utility score (+0.027 ± 0.012; P = .04). In the PDFF responders group, several improvements were seen in the HQRL scores by the end of week 36: Physical Functioning (mean ± standard error: +6.76 ± 2.62; P = .012), Bodily Pain (+9.39 ± 3.18; P = .010), PCS (+2.99 ± 0.76; P = .00062), and Vitality (+4.04 ± 2.28; P = .056), with an overall improvement in health-related scores. These changes were more consistent in the population of patients whose SHGB levels (a hepatic marker of resmetirom exposure) were increased by at least 88% from baseline. The Mental Component Score (MCS) was improved in patients with at least 5% weight reduction at the end of week 12 (+10.3 + 3.9; P = 0.018), and those who did not lose weight did not report any improvement in the MCS (+1.16 + 1.60; P = 0.24) with a significant difference between the two groups (P=0.037) which was continued until week 36. The results were higher in patients with clinical improvement in MRI-PDFF by more than 30% by week 12, which was associated with higher improvement rates in Physical Functioning and PCS scores (P<0.05). When compared to non-responders on liver biopsy, responders (with at least two-point reduction in NAS) were found to have more significant improvement in HQRL scores, including Mental Health and SF-6D utility score, and the responders with a decrease of at least one-point fibrosis had improved general health when compared to non-responders and those on placebo. The above results have shown that resmetirom is more beneficial than a placebo in improving the quality of life in NASH patients. However, the long-term sustainment of the improvements has to be confirmed by further studies.

However, the study's sample size was small, with the majority of patients being in the early stage of the disease, excluding the impact of HQRL on advanced stages such as cirrhosis. Also, HQRL scores being self-reported, the risk of recall bias cannot be excluded in concluding and additional studies focusing on disease-specific HQRL instruments such as the Chronic-Liver Disease Questionnaire and other significant co-morbidities have to be considered.

Cost-Effective Analysis

Resmetirom was found to increase the QALYs by 1.24 times in patients with NASH. However, it was estimated that resmetirom treatment costs around US$66,764 per patient. At a willingness to pay threshold of US $ 100,00, resmetirom could be a cost-saving measure as the incremental cost-effectiveness ratio was US $ 53,929 per QALY gained. Javanbakt et al. developed the first economic model exploring the cost-effectiveness of resmetirom compared to placebo in 2023, including all disease-relevant health states and complications relying on clinical data obtained from the phase-II resmetirom trial [18]. The results revealed that resmetirom decreases the development of around 87 new cases of decompensated cirrhosis, 59 cases of hepatocellular carcinoma, and 30 cases of liver transplantation per 1000 patients treated with resmetirom compared to placebo. Up to the daily threshold value of US$72.00, resmetirom remained cost-effective at a Willingness-to-Pay threshold (WTP) of US$100,000. The following analyses were made in the review to estimate the real-life monetary burden for resmetirom treatment.

Base-case analysis: During the cost-utility analysis focusing on cost per QALY when resmetirom was introduced to treat NASH and fibrosis, the cost per patient was estimated to be US$348,432 over the lifetime at a WTP threshold of US$100,000 per QALY as compared to placebo which is US$66,764 cheaper than resmetirom costing around US$281,668. However, the QALYs gained with resmetirom (12.75) was higher than that of placebo (11.52) over a lifetime with an incremental QALY gain of 1.24, leading to an incremental cost-effectiveness ratio of US$53,929 per QALY, which is lower than the WTP US$100,000 per QALY. With the increasing WTP threshold, the probability of resmetirom being cost-effective increases with an 86.20% probability of being cost-effective at US$100,000 WTP

One-way sensitive analysis: The one-way deterministic sensitivity analysis (DSA) was estimated based on the 95% confidence intervals or + 20% variations. Net monetary benefit (NMB) is the value of intervention in terms of monetary benefit, which could be calculated as (incremental benefit × threshold) - incremental cost. Resmetirom had a net monetary benefit of US$60,063, and the model result drivers are the proportion of patients achieving no changes in fibrosis at week 36

Threshold analysis: a threshold analysis was performed to estimate the cost-effectiveness of resmetirom at a daily price with US$50,000, US$100,00, and US$150,000 WTP thresholds. The results revealed that resmetirom would still be cost-effective with a daily price of US$50.35 at a WTP of US$50,000, US$72.00 at a WTP of US$100,000, and US$93.64 at a US$150,000 WTP threshold meaning the drug would be cost-effective when the maximum daily price reaches the values mentioned above according to WTP.

Scenario analysis: Exploring the impact of alternative approaches estimating the cardiovascular risk using the resmetirom phase II trial patient data, two separate scenario analyses were performed where a pooled cardiovascular risk for both resmetirom and placebo arms was used assuming NAFLD and NASH as independent risk factors after controlling the baseline risk using phase II trial patient-level data in one scenario. In the second scenario, the average 10-year cardiovascular risk in patients above 40 years in both arms was used based on phase-II trial patient-level data. The results were consistent with base-case analysis with incremental cost-effectiveness ratios of US$58,355 and US$59,716 per QALY gained in the above scenarios, respectively.

However, this model assumes that the progression of the disease is linear without accounting for the variations in the progression of the disease for each individual, which is an inherent flaw in the approach. This study also did not take into account the fact that NASH can co-exist with other highly chronic conditions such as T2DM and obesity, with high prevalence rates affecting the long-term outcomes adversely with higher complication rates and progression to the advanced stage of fibrosis impacting the cost-effectiveness analyses results. Notably, the fact that the treatment benefits extend beyond their action on hepatocytes, there is an unmet need for economic models of NASH considering the cardiovascular outcomes associated with other co-morbidities. This model only estimated the short-term cardiovascular risk; therefore, the study did not thoroughly analyze the prolonged effects of resmestirom on reducing LDL-C and cardiovascular risk levels. This study also estimated a 95% fixed adherence to treatment in real life and calculated the data to 30 years from the data obtained from 36 weeks.

Limitations

This study was limited by the number of available clinical trials, and the long-term credibility has not been evaluated yet. Also the lower sample size and shorter period of follow-ups cannot analyze the full spectrum of events consequent to the use of resmetirom and the sustainability of achieved results requiring to perform additional long-term trials with a larger population. Also, the practical aspects such as the rate of progression of liver fibrosis, loss of adherence to treatment, and other existent co-morbid conditions were not taken into consideration in the above studies. Hence, final conclusions cannot be drawn at this point without further studies. However, this study has analyzed all the available information on resmetirom to date thoroughly focusing on all the required aspects of NASH.

## Conclusions

After extensively analyzing the collected data on resmetirom and its actions on patients with NASH along with the other properties of cost-effectiveness, and improved QALY, it can be concluded that resmetirom holds strong potential for use in the treatment of NASH. Due to its additional benefits of lowering cardiovascular risk factors and improving patient-reported outcomes, resmetirom can be undoubtedly beneficial to the patients and the result can be best achieved when traditional measures such as weight loss are combined with resmetirom. However, a vast amount of research is yet required to be done in multiple participants with consideration to various conditions such as progression of fibrosis, existing co-morbidities, risk of recall bias in self-reported outcomes, and sustainment of achieved results. Though resmetirom has shown to be a promising drug in treating NASH, it is still under investigation and not an established treatment and can be expected to be brought into clinical use in the near future.
